# Creating machine learning models that interpretably link systemic inflammatory index, sex steroid hormones, and dietary antioxidants to identify gout using the SHAP (SHapley Additive exPlanations) method

**DOI:** 10.3389/fimmu.2024.1367340

**Published:** 2024-05-01

**Authors:** Shunshun Cao, Yangyang Hu

**Affiliations:** ^1^ Pediatric Endocrinology, Genetics and Metabolism, The Second Affiliated Hospital and Yuying Children’s Hospital of Wenzhou Medical University, Wenzhou, Zhejiang, China; ^2^ Reproductive Medicine Center, Obstetrics and Gynecology, The Second Affiliated Hospital and Yuying Children’s Hospital of Wenzhou Medical University, Wenzhou, Zhejiang, China

**Keywords:** gout, systemic inflammation index, sex steroid hormones, dietary antioxidants, NHANES

## Abstract

**Background:**

The relationship between systemic inflammatory index (SII), sex steroid hormones, dietary antioxidants (DA), and gout has not been determined. We aim to develop a reliable and interpretable machine learning (ML) model that links SII, sex steroid hormones, and DA to gout identification.

**Methods:**

The dataset we used to study the relationship between SII, sex steroid hormones, DA, and gout was from the National Health and Nutrition Examination Survey (NHANES). Six ML models were developed to identify gout by SII, sex steroid hormones, and DA. The seven performance discriminative features of each model were summarized, and the eXtreme Gradient Boosting (XGBoost) model with the best overall performance was selected to identify gout. We used the SHapley Additive exPlanation (SHAP) method to explain the XGBoost model and its decision-making process.

**Results:**

An initial survey of 20,146 participants resulted in 8,550 being included in the study. Selecting the best performing XGBoost model associated with SII, sex steroid hormones, and DA to identify gout (male: AUC: 0.795, 95% CI: 0.746- 0.843, accuracy: 98.7%; female: AUC: 0.822, 95% CI: 0.754- 0.883, accuracy: 99.2%). In the male group, The SHAP values showed that the lower feature values of lutein + zeaxanthin (LZ), vitamin C (VitC), lycopene, zinc, total testosterone (TT), vitamin E (VitE), and vitamin A (VitA), the greater the positive effect on the model output. In the female group, SHAP values showed that lower feature values of E2, zinc, lycopene, LZ, TT, and selenium had a greater positive effect on model output.

**Conclusion:**

The interpretable XGBoost model demonstrated accuracy, efficiency, and robustness in identifying associations between SII, sex steroid hormones, DA, and gout in participants. Decreased TT in males and decreased E2 in females may be associated with gout, and increased DA intake and decreased SII may reduce the potential risk of gout.

## 1 Introduction

Gout is a metabolic disease caused by the accumulation of urate crystals in the joints of the limbs as well as in the tissues and organs (1). Hyperuricemia is defined when the blood uric acid (UA) concentration is greater than 7.0 mg/dl for men and 6.0 mg/dl for women, the blood is oversaturated with UA, and urate crystals begin to crystallize, making hyperuricemia the most critical risk factor for gout (2). A recent study reported that population-based epidemiologic surveys in Asia, Europe, and North America showed a prevalence of gout ranging from 0.6% to 2.9%, with adult prevalence ranging from 0.68% to 3.90%, and that gout is increasingly becoming a major health problem that is difficult to avoid (3). Whereas in the past gout had been considered to be merely a disorder of purine metabolism, there is now a greater tendency to view it as a multifactorial autoinflammatory disease in which hyperuricemia alone is not sufficient to cause gout. Therefore, an in-depth understanding of the pathophysiology of gout is particularly important (4).

Studies have shown that UA acts as an antioxidant that protects by scavenging oxygen free radicals and preventing lipid peroxidation, accounting for 30 to 50 percent of the body’s normal antioxidant capacity (5). At present, many studies have shown that gout is closely related to oxidative stress and inflammation (6, 7). Antioxidants are effective in breaking down harmful oxygen free radicals generated by oxidative stress, and dietary antioxidants (DA) are one of the most important sources of exogenous antioxidants that humans are exposed to in their daily lives (8). Common DA includes antioxidant vitamins, antioxidant minerals, lycopene, flavonoids, resveratrol, and anthocyanins (9). The systemic inflammation index (SII) is calculated by multiplying the platelet count by the neutrophil count divided by the lymphocyte count, which can assess the systemic inflammatory state of the body (10). The Composite Dietary Antioxidant Index (CDAI) is a highly efficient and accurate nutritional tool for assessing the overall antioxidant properties of a diet and consists of a composite score of six dietary antioxidants (11). However, no study has confirmed the relationship between SII, DA, and gout. A Mendelian randomization study showed that higher testosterone levels based on genetic prediction in males were associated with a lower risk of type 2 diabetes, gout, and celiac disease while noting that sex hormones, sex hormone-binding globulin, and metabolic diseases are strongly associated (12). At present, most of the relevant studies use traditional statistical analysis methods, whereas our use of machine learning (ML) may be more conducive to accurately determining the relationship between SII, sex steroid hormones, DA, and gout.

ML is one of the most intelligent features and cutting-edge research areas of artificial intelligence (AI), which provides a set of tools to classify and predict based on patterns observed in medical data, as well as the study of computer algorithms that can be automatically improved empirically (13, 14). State-of-the-art ML algorithms significantly outperform traditional predictive models in predicting gout risk using large and complex datasets. ML suits large-sample, multi-correlation feature studies with the National Health and Nutrition Examination Survey (NHANES) dataset. This study aimed to develop six ML models for gout identification using NHANES data. The best-performing model was selected and used SHapley Additive exPlanations (SHAP)-based ML visualization to determine the contribution of SII, sex steroid hormones, and DA to gout identification, enhancing the potential for early prevention and intervention.

## 2 Materials and methods

### 2.1 Data selection and study design

Data for this study were obtained from the NHANES dataset. As described by Zhao M et al, NHANES (2013–2016) is a cross-sectional survey program using complex random probability samples with a multi-stage stratified design conducted by the National Center for Health Statistics of the Centers for Disease Control and Prevention (15). The survey covers demographics, lifestyle, anthropometric, laboratory analysis, questionnaire interviews, and dietary data. The NHANES study plan was approved by the National Center for Health Statistics (NCHS) Ethics Review Board, Approval No. #2011-17 (16). Written informed consent was obtained from all participants for this study including the Home Interview Consent Form, Consent for Specimen Storage and Continuing Studies, and Consent for Examination at the Mobile Examination Center.

Because NHANES 2013 to 2016 has complete data on male and female sex steroid hormones, we chose these 2 cycles as the source of data for our study. We collected a total of 20,146 male and female samples for the initial survey. Inclusion criteria were (1) age ≥20 years (2); participation in dietary interviews and sex steroid hormone testing; and (3) full participant gout information. Exclusion criteria were (1) age < 20 years (2); missing self-report of gout (3); pregnancy in female participants; and (4) missing information on SII, DA, sex steroid hormones, and other important variables. Finally, 8550 participants were included in the study, including 4160 males and 4390 females.

### 2.2 Definition of gout

The 2013-2016 NHANES data that we initially included in the study was an all-age population, and the age limit for those who conducted the Interview Gout Questionnaire was 20 years of age and older; therefore, we needed to exclude those under 20 years of age. We used self-reported personal interview data from the MCQ160N questionnaire (**Supplementary Material 1**) to categorize whether study participants had gout. Participants were asked, “Has a doctor ever told you that you had gout?” Choice code 1 indicated gout, code 2 indicated no gout, code 7 indicated refusal to answer, and code 9 indicated not sure.

### 2.3 Determination of sex steroid hormones

Serum specimens were separated after venous blood collection, stored at -30°C, and shipped to the Centers for Disease Control and Prevention for uniform testing by the Laboratory Sciences Division of the National Center for Environmental Health. Serum total testosterone (TT) levels and estradiol (E2) concentrations were determined by isotope dilution liquid chromatography-tandem mass spectrometry (ID-LC-MS/MS). The sex hormone-binding globulin (SHBG) assay is based on chemiluminescence measurements, and readings are compared to instrument- and lot-specific calibration curves. Detailed analytical methods are described in the NHANES Laboratory Procedures Manual. The lower detection limits for TT, E2, and SHBG were 0.75 ng/dL, 2.994 pg/mL, and 0.800 nmol/L, respectively.

### 2.4 Collection of demographic and other laboratory data

The demographic profiles we included in the study included gender, age, race, educational attainment, and family poverty income ratios (PIR). Tobacco use, alcohol consumption, hypertension, and diabetes were determined by an interview questionnaire, with at least 100 cigarettes smoked in a lifetime as definitive tobacco use. Total dietary calories and total lycopene intake data for 2 days were obtained through nutrition interviews. They got BMI and waist measurement data from body measurements. Other laboratory tests include triglycerides (TG), hemoglobin (Hb), UA, high-density lipoprotein (HDL), low-density lipoprotein (LDL), apolipoprotein B (ApoB), glycosylated hemoglobin (GHB) and 1, 25-hydroxyvitamin D3 (VitD).

### 2.5 Definition of SII, DA, and CDAI

According to the method of Wei C et al. (10), the calculation formula of SII is platelet count multiplied by neutrophil count divided by lymphocyte count. Dietary data were obtained through two dietary recall interviews. The NHANES-assisted dietary interview system calculated the energy and nutrient intakes obtained from the number and type of food, beverage, and water within 24 hours before the interview. The six antioxidants we extracted included vitamin A (VitA), vitamin C (VitC), vitamin E (VitE), zinc, selenium (Se), and lutein + zeaxanthin (LZ). DA in this study mainly refers to six antioxidants and lycopene. The CDAI was calculated by subtracting the mean value from each of the six antioxidants and dividing it by the standard deviation to standardize and add them together (17).

### 2.6 Feature engineering and model development

To avoid overfitting, the participant data used for ML development were subjected to principal component analysis (PCA), which ultimately consisted of 29 features and 1 label, of which 4 features were categorical variables and the remaining 25 features were continuous variables. Variables with missing values greater than 20% were directly deleted, and variables with missing values less than 20% were interpolated for missing values using the ML-based random forest (RF) algorithm (18). You J et al. found that RF interpolation significantly outperformed other interpolation methods in terms of both average prediction error and consistency correlation coefficient after comparing multiple missing value interpolation methods (19). We randomly split the data into the 80% training set (male group n=3328, female group n=3512) and the 20% test set (male group n=832, female group n=878), subjected the training set to Synthetic Minority Over-Sampling Technique (SMOTE) to deal with data imbalance, normalized the feature data using a MinMaxScaler, and represented the categorical variables using one-hot coding (20, 21).

We used six ML models, support vector machine (SVM), Light Gradient Boosting Machine (LGBM), RF, Gradient Boosting Decision Trees (GBDT), Extreme Gradient Boosting (XGBoost), and Category Boosting (CatBoost), to identify gout associated with SII, sex steroid hormones, and DA. To optimize the performance of each ML algorithmic model, we tuned the hyperparameters using a grid search (22). We adopt the method of Cao Y et al. using 5-fold cross-validation, where the training set data is divided into 5 subsets, and each model is trained on 4 of the partitioned data and validated on the remaining partitioned data to calculate the average performance metrics (23). Different ML algorithms have their adaptations to the data and analysis, so we used the training set to develop six ML models and to verify the reliability, accuracy, and robustness of the models we used the test set (24). Important reference metrics used to evaluate the performance and generalization ability of ML models include the area under the ROC curve (AUC), accuracy, precision, recall, F1 score, brier score, and the area under the P-R curve (AP), as described by Goodswen SJ et al. (25). After summarizing seven metrics representing model performance and generalization ability for each ML model, the overall best algorithmic model for identifying gout was selected and the model was interpreted using SHAP. SHAP is one of the most commonly used *post hoc* interpretability tools. It enhances the user’s trust in the model by calculating the marginal contribution of each feature to the model’s output, interpreting the model both globally and locally, and giving the process of prediction and decision-making provided by the model (26).

### 2.7 Statistical analysis

In this study, the demographic characteristics of study participants with and without gout were described and grouped by gender. We used the Wilcoxon rank sum test for complex survey samples to compare differences between two groups for continuous variables and the Rao & Scott second-order corrected chi-square test to compare differences between groups for categorical variables. The Kruskal-Wallis test was used for comparison of data between quartile groups. Depending on the distribution of the data, continuous variables are expressed as mean ± standard deviation or median and quartiles (25%, 75%), while categorical variables are expressed as frequencies (percentages). ML model performance was discriminated using the AUC, accuracy, precision, recall, F1 score, brier score, and the AP for each model. All data were analyzed using R software version 4.3.1 and Python software version 3.11.5, respectively. The R packages used are gtsummary, survey, haven, tableone, plyr, dplyr, tidyverse, arsenal, missForest. ML was primarily analyzed using the scikit-learn 1.2.2 library. P-values less than 0.05 were considered statistically different.

## 3 Results

### 3.1 Baseline characteristics of participants

**Figure 1:**
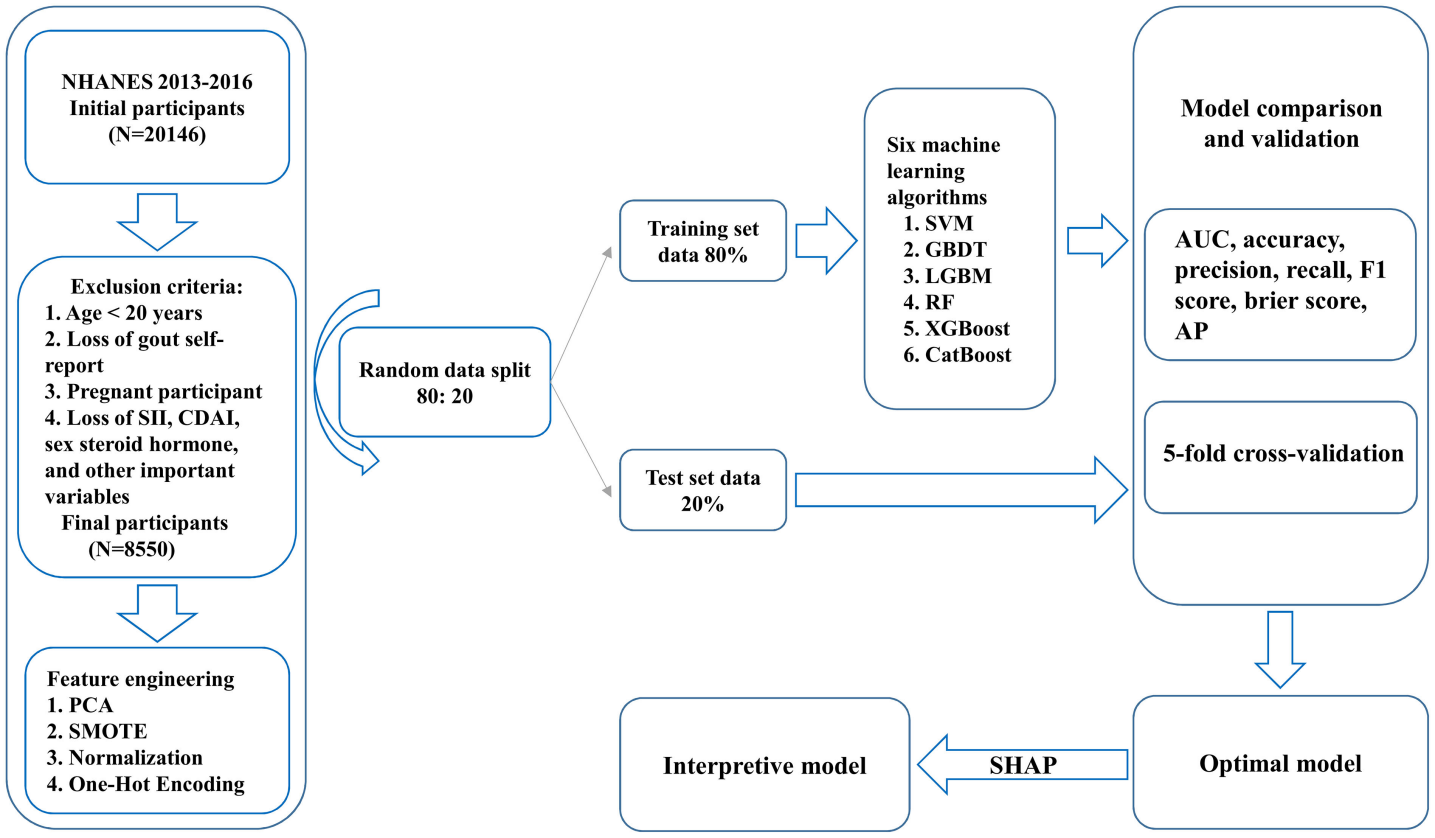
Participant selection and study design flowchart.

**Figure 1** illustrates the baseline process of participant selection and study design. A total of 8550 participants were included in the study, and the demographic characteristics of participants diagnosed with and without gout are summarized in **Table 1**. Participants were 48.65% (n=4160) male and 51.35% (n=4390) female, with an overall gout prevalence of 4.32% (n=369), with 70.19% male and 29.81% female, which is a significant gender difference (P<0.05). In the overall population gout patients were more likely to be male, older, have higher TT levels, lower E2 levels, lower SHBG levels, higher TG levels, higher UA levels, lower HDL levels, higher GHB levels, diabetes, Non-Hispanic White, alcohol consumption, tobacco use, obesity, and hypertension (P<0.05).

**Table 1:** Basic characteristics of NHANES participants by gout group.

Characteristic	Overall	Gout group		
	N = 8550 (100%)	Yes, N = 369 (4.32%)	No, N = 8181 (95.68%)	P-value
Gender				<0.001
Female	4,390 (51.35%)	110 (29.81%)	4,280 (52.32%)	
Male	4,160 (48.65%)	259 (70.19%)	3,901 (47.68%)	
Age (years)	48.00 (33.00, 61.00)	62.00 (54.00, 70.00)	47.00 (33.00, 60.00)	<0.001
PIR	2.87 (1.43, 5.00)	2.96 (1.28, 5.00)	2.86 (1.43, 5.00)	0.800
TT (ng/dL)	64.43 (19.40, 389.00)	260.00 (25.52, 393.95)	54.22 (19.20, 388.00)	0.037
E2 (pg/mL)	23.00 (12.50, 36.00)	21.43 (12.08, 28.25)	23.10 (12.50, 36.60)	0.003
SHBG (nmol/L)	48.58 (32.42, 74.84)	45.62 (31.95, 59.69)	48.69 (32.46, 75.30)	0.016
TG (mmol/L)	1.07 (0.73, 1.66)	1.69 (0.97, 2.45)	1.06 (0.72, 1.61)	<0.001
Hb (g/dL)	14.20 (13.30, 15.20)	14.30 (13.30, 15.25)	14.20 (13.30, 15.20)	0.600
UA (umol/L)	315.20 (261.70, 374.70)	380.70 (321.20, 463.90)	315.20 (261.70, 368.80)	<0.001
HDL (mmol/L)	1.32 (1.09, 1.63)	1.16 (0.91, 1.42)	1.34 (1.09, 1.66)	<0.001
LDL (mmol/L)	2.87 (2.25, 3.52)	2.52 (1.86, 3.37)	2.87 (2.28, 3.52)	0.049
VitD (nmol/L)	64.60 (46.90, 83.10)	62.73 (41.50, 84.81)	64.70 (47.10, 83.00)	0.400
Total calories (kcal)	1,956 (1,511, 2,503)	1,939 (1,428, 2,470)	1,957 (1,514, 2,504)	0.500
ApoB (mg/dL)	90.00 (75.00, 108.00)	91.57 (76.00, 114.00)	90.00 (75.00, 107.00)	0.200
GHB (%)	5.50 (5.20, 5.80)	5.70 (5.50, 6.30)	5.40 (5.20, 5.80)	<0.001
Diabetes group				<0.001
No	7,123 (85.73%)	230 (65.34%)	6,893 (86.63%)	
Yes	1,186 (14.27%)	122 (34.66%)	1,064 (13.37%)	
Age group				<0.001
20-49 (years)	4,344 (50.81%)	56 (15.18%)	4,288 (52.41%)	
50-79 (years)	3,711 (43.40%)	272 (73.71%)	3,439 (42.04%)	
80 and over (years)	495 (5.79%)	41 (11.11%)	454 (5.55%)	
Race				0.001
Non-Hispanic White	3,332 (38.97%)	164 (44.44%)	3,168 (38.72%)	
Non-Hispanic Black	1,658 (19.39%)	86 (23.31%)	1,572 (19.22%)	
Mexican American	1,387 (16.22%)	41 (11.11%)	1,346 (16.45%)	
Other/multiracial	1,187 (13.88%)	45 (12.20%)	1,142 (13.96%)	
Other Hispanic	986 (11.53%)	33 (8.94%)	953 (11.65%)	
Alcohol consumption				0.064
1-5 (drinks/month)	4,076 (50.05%)	181 (50.99%)	3,895 (50.01%)	
6-10 (drinks/month)	614 (7.54%)	16 (4.51%)	598 (7.68%)	
More than 10 (drinks/month)	1,055 (12.96%)	70 (19.71%)	985 (12.65%)	
Non- drinker	2,398 (29.45%)	88 (24.79%)	2,310 (29.66%)	
Tobacco use				<0.001
Never smoker	4,916 (57.56%)	162 (43.90%)	4,754 (58.17%)	
Former smoker	2,004 (23.46%)	161 (43.63%)	1,843 (22.55%)	
Current smoker	1,621 (18.98%)	46 (12.47%)	1,575 (19.28%)	
BMI group				<0.001
Underweight(<18.5 kg/m^2^)	122 (1.44%)	2 (0.55%)	120 (1.48%)	
Normal(18.5 to <25 kg/m^2^)	2,244 (26.46%)	52 (14.29%)	2,192 (27.00%)	
Overweight(25 to <30 kg/m^2^)	2,741 (32.32%)	105 (28.85%)	2,636 (32.47%)	
Obese(30 or greater kg/m^2^)	3,375 (39.79%)	205 (56.31%)	3,170 (39.05%)	
Education attainment				0.500
Less Than 9th Grade	819 (9.58%)	32 (8.67%)	787 (9.62%)	
9-11th Grade	1,053 (12.32%)	53 (14.36%)	1,000 (12.23%)	
High School Grad/GED	1,908 (22.32%)	95 (25.75%)	1,813 (22.17%)	
Some College or AA degree	2,607 (30.50%)	112 (30.35%)	2,495 (30.51%)	
College Graduate or above	2,160 (25.27%)	77 (20.87%)	2,083 (25.47%)	
Hypertension				<0.001
No	4,935 (61.02%)	72 (20.81%)	4,863 (62.81%)	
Yes	3,153 (38.98%)	274 (79.19%)	2,879 (37.19%)	
CDAI	-0.48 (-2.17, 1.69)	-0.60 (-2.23, 1.32)	-0.48 (-2.17, 1.72)	0.300
SII	460.27 (333.18, 633.15)	474.70 (333.43, 681.89)	459.67 (333.00, 630.50)	0.300
Lycopene (mcg)	1,897.00 (551.00, 5,596.50)	1,893.32 (531.50, 5,330.59)	1,895.50 (551.00, 5,608.24)	>0.900

PIR, family poverty income ratios; TT, total testosterone; E2, estradiol; SHBG, sex hormone-binding globulin; TG, triglycerides; Hb, hemoglobin; UA, uric acid; HDL, high-density lipoprotein; LDL, low-density lipoprotein; VitD, 1, 25-hydroxyvitamin D3; ApoB, apolipoprotein B; GHB, glycosylated hemoglobin; CDAI, composite dietary antioxidant index; SII, systemic immune-inflammatory index.

### 3.2 Comparison of variables among weighted participants grouped by quartiles of UA

We categorized males and females into Q1-Q4 groups based on quartiles of UA levels, respectively. **Table 2** shows that among the male participants, the differences between the Q2, Q3, and Q4 groups compared to the Q1 group were statistically significant for all variables except SII, Lycopene, zinc, Se, and LZ (P<0.05). **Table 3** shows that among the female participants, the differences between the Q2, Q3, and Q4 groups compared to the Q1 group were statistically significant for all variables except SII, lycopene, VitA, Se, and LZ (P<0.05).

**Table 2:** Comparison of variables in weighted male participants grouped by serum uric acid quartiles.

Characteristic	Q1 N = 8,596,521	Q2 N = 20,890,675	Q3 N = 30,920,660	Q4 N = 36,166,082	P-value
UA (umol/L)	238 (226, 256)	297 (286, 309)	345 (333, 363)	422 (399, 458)	<0.001
TT (ng/dL)	419 (302, 575)	428 (330, 557)	417 (306, 523)	351 (273, 461)	<0.001
E2 (pg/mL)	22 (17, 28)	23 (18, 29)	24 (19, 29)	25 (19, 30)	<0.001
SHBG(nmol/L)	47 (32, 65)	43 (32, 58)	37 (27, 53)	34 (24, 47)	<0.001
CDAI	0.9 (-1.2, 3.2)	0.9 (-1.2, 3.4)	0.2 (-1.7, 2.5)	0.1 (-1.8, 2.2)	0.015
SII	448 (313, 589)	435 (306, 613)	434 (315, 609)	450 (333, 610)	0.500
Lycopene (mcg)	2,245 (574, 6,430)	2,208 (686, 6,009)	2,515 (625, 7,110)	2,029 (607, 5,908)	0.300
VitA (mcg)	683 (408, 1,028)	646 (390, 984)	550 (346, 829)	507 (325, 792)	<0.001
VitC (mg)	67 (32, 124)	75 (31, 128)	59 (29, 113)	53 (24, 108)	0.035
Zinc (mg)	12.7 (9.1, 16.2)	12.6 (9.4, 17.0)	12.1 (8.9, 15.7)	11.6 (8.1, 16.0)	0.075
Se (mcg)	133 (97, 164)	129 (96, 169)	123 (92, 160)	123 (94, 164)	0.300
LZ (mcg)	947 (504, 1,563)	973 (521, 1,904)	885 (458, 1,712)	832 (482, 1,467)	0.079

VitA, vitamin A; VitC, vitamin C; Se, selenium; LZ, lutein + zeaxanthin.

**Table 3:** Comparison of variables in weighted female participants grouped by serum uric acid quartiles.

Characteristic	Q1 N = 41,107,484	Q2 N = 28,952,815	Q3 N = 19,407,319	Q4 N = 11,697,967	P-value
UA (umol/L)	226 (202, 250)	286 (280, 303)	339 (327, 357)	410 (393, 440)	<0.001
TT (ng/dL)	19 (13, 27)	21 (14, 29)	21 (14, 30)	18 (13, 26)	0.002
E2 (pg/mL)	28 (5, 94)	23 (6, 72)	14 (6, 60)	9 (5, 26)	<0.001
SHBG (nmol/L)	75 (51, 108)	64 (43, 90)	53 (33, 80)	52 (34, 74)	<0.001
CDAI	-0.86 (-2.39, 1.09)	-1.06 (-2.64, 0.87)	-1.32 (-2.68, 0.31)	-1.44 (-2.79, 0.35)	0.033
SII	476 (341, 649)	474 (341, 626)	486 (342, 703)	497 (350, 681)	0.300
Lycopene (mcg)	1,773 (558, 5,085)	1,629 (436, 5,114)	1,561 (429, 4,742)	1,380 (420, 4,775)	0.200
VitA (mcg)	524 (329, 785)	515 (299, 766)	484 (318, 692)	469 (323, 784)	0.140
VitC (mg)	61 (30, 111)	56 (25, 104)	55 (27, 93)	52 (25, 94)	0.019
Zinc (mg)	9.1 (6.7, 11.7)	8.7 (6.3, 11.5)	8.3 (6.3, 11.4)	8.0 (6.2, 11.1)	0.007
Se (mcg)	95 (72, 121)	91 (68, 118)	91 (69, 123)	88 (66, 115)	0.130
LZ (mcg)	901 (472, 2,012)	819 (438, 1,908)	830 (467, 1,609)	858 (418, 1,841)	0.400

### 3.3 Comparison of model performance of ML to identify gout

**Figure 2:**
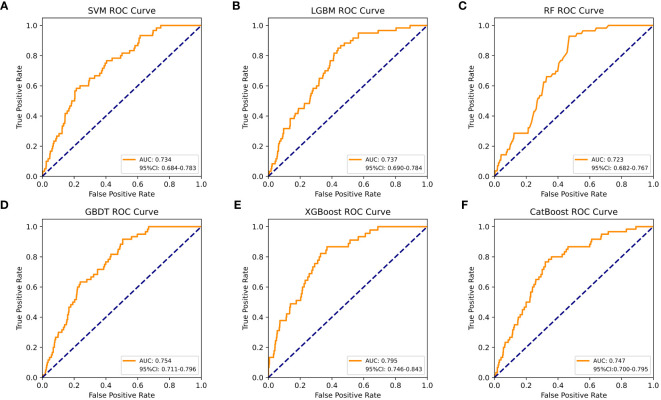
The AUC comparison of six ML models in the male group. **(A)** SVM, **(B)** LGBM, **(C)** RF, **(D)** GBDT, **(E)** XGBoost, and **(F)** CatBoost.

**Figure 3:**
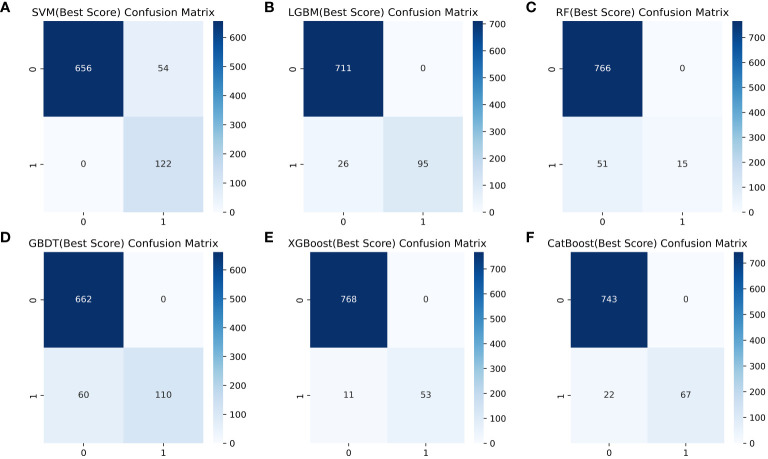
Comparison of six ML model confusion matrices for the male group. **(A)** SVM, **(B)** LGBM, **(C)** RF, **(D)** GBDT, **(E)** XGBoost, and **(F)** CatBoost.

**Figure 4:**
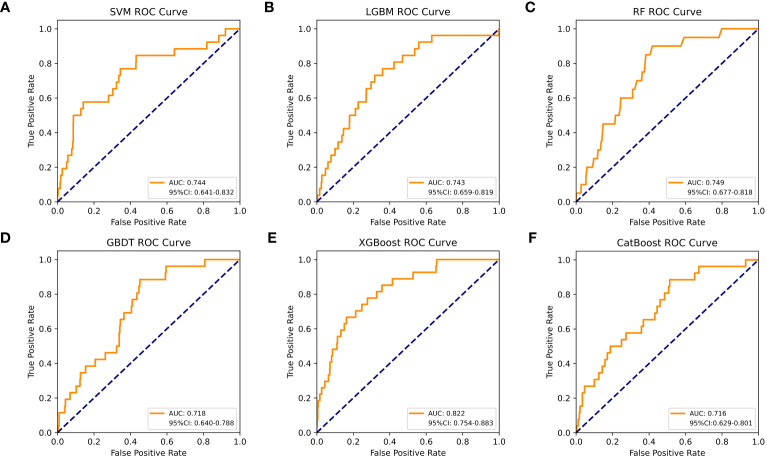
The AUC comparison of six ML models in the female group. **(A)** SVM, **(B)** LGBM, **(C)** RF, **(D)** GBDT, **(E)** XGBoost, and **(F)** CatBoost.

**Figure 5:**
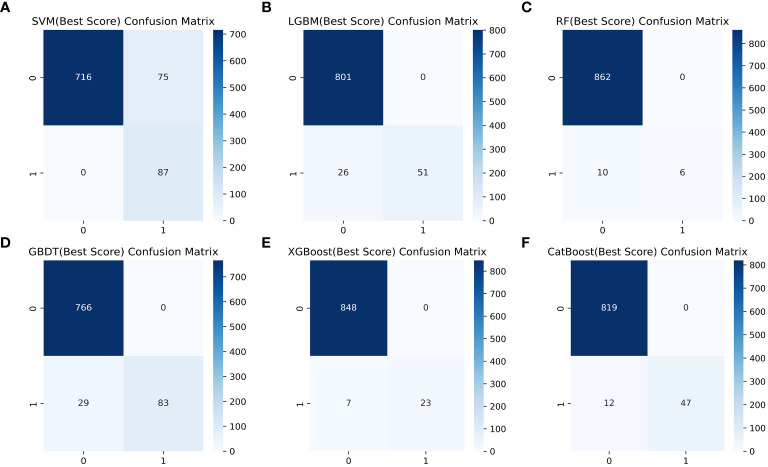
Comparison of six ML model confusion matrices for the female group. **(A)** SVM, **(B)** LGBM, **(C)** RF, **(D)** GBDT, **(E)** XGBoost, and **(F)** CatBoost.

**Table 4** shows a summary of the model performance metrics of the six ML models for identifying gout in different gender groups based on the test set. In the male group, XGBoost showed optimal AUC performance with an AUC (95%CI) of 0.795 (0.746-0.843) based on the test set data (**Figure 2**). The XGBoost model demonstrated superior discrimination ability, achieving an accuracy of 98.7%, precision of 100%, recall of 82.8%, F1 score of 0.906, brier score of 0.067, and AP of 0.238 based on the test set data (**Figure 3**). The XGBoost algorithm demonstrated superior performance in terms of AUC (95%CI) with a value of 0.822 (0.754-0.883) based on the test set data, specifically within the female group (**Figure 4**). The XGBoost model also demonstrated superior discrimination ability, achieving an accuracy of 99.2%, precision of 100%, recall of 76.7%, F1 score of 0.868, brier score of 0.035, and AP of 0.207 based on the test set data (**Figure 5**).

**Table 4:** Performance evaluation of six machine learning models for different gender groups (Male/Female).

Characteristics	SVM	LGBM	RF	GBDT	XGBoost	CatBoost
AUC	0.734/0.744	0.737/0.743	0.723/0.749	0.754/0.718	0.795/0.822	0.747/0.716
(95% CI)	(0.684-0.783/ 0.641-0.832)	(0.690-0.784/ 0.659-0.819)	(0.682-0.767/ 0.677-0.818)	(0.711-0.796/ 0.640-0.788)	(0.746-0.843/ 0.754-0.883)	(0.700-0.795/ 0.629-0.801)
Accuracy	0.935/0.915	0.969/0.970	0.939/0.989	0.928/0.967	0.987/0.992	0.974/0.986
Precision	0.693/0.537	1.000/1.000	1.000/1.000	1.000/1.000	1.000/1.000	1.000/1.000
Recall	1.000/1.000	0.785/0.662	0.227/0.375	0.647/0.741	0.828/0.767	0.753/0.797
F1 score	0.819/0.699	0.880/0.797	0.370/0.545	0.786/0.851	0.906/0.868	0.859/0.887
Brier score	0.140/0.109	0.105/0.060	0.083/0.036	0.112/0.077	0.067/0.035	0.094/0.053
AP	0.167/0.150	0.158/0.084	0.135/0.101	0.170/0.081	0.238/0.207	0.175/0.090

SVM, support vector machine; LGBM, light gradient boosting machine; RF, random forest; GBDT, gradient boosting decision tree; XGBoost, extreme gradient boosting; CatBoost, category boosting; AUC, The area under the ROC curve; AP, The area under the P-R curve.

### 3.4 SHAP interpretation and feature importance visualization

**Figure 6:**
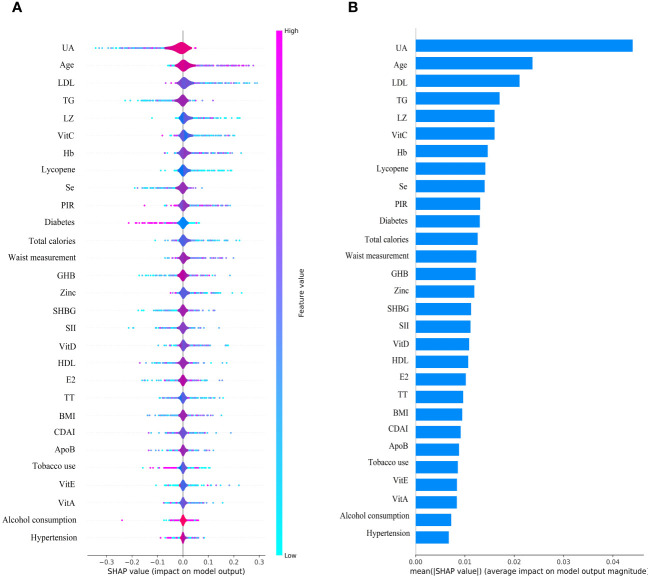
Male group XGBoost ML model SHAP plot. **(A)** SHAP summary plot, **(B)** feature importance.

**Figure 7:**
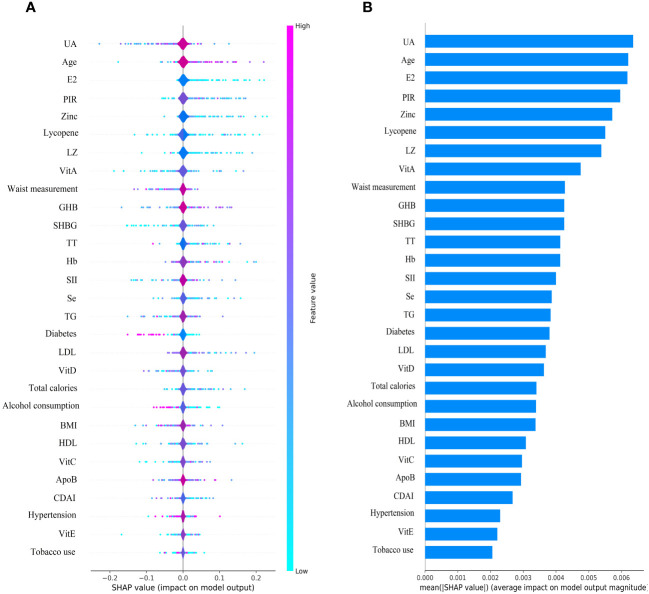
Female group XGBoost ML model SHAP plot. **(A)** SHAP summary plot, **(B)** feature importance.

The SHAP plot illustrates the impact of all features in the XGBoost model on identifying gout within the test dataset. The importance feature by SHAP showed that UA was the most important feature for the identification of gout by the XGBoost model in both male and female groups. Using the SHAP summary plot, we found that in the male group, the lower the feature values of LZ, VitC, lycopene, zinc, TT, VitE, and VitA, the greater the positive impact on the model output, while the lower the feature values of UA, Se, SHBG, SII, and E2, the greater the negative impact on the model output (**Figure 6**). In the female group, SHAP values showed that lower feature values of E2, Zinc, Lycopene, LZ, TT, and Se had a greater positive effect on model output. In comparison, lower feature values of VitA, SHBG, and SII had a greater negative effect on model output (**Figure 7**).

### 3.5 SHAP interpretability of individual decisions in the XGBoost model

**Figure 8:**
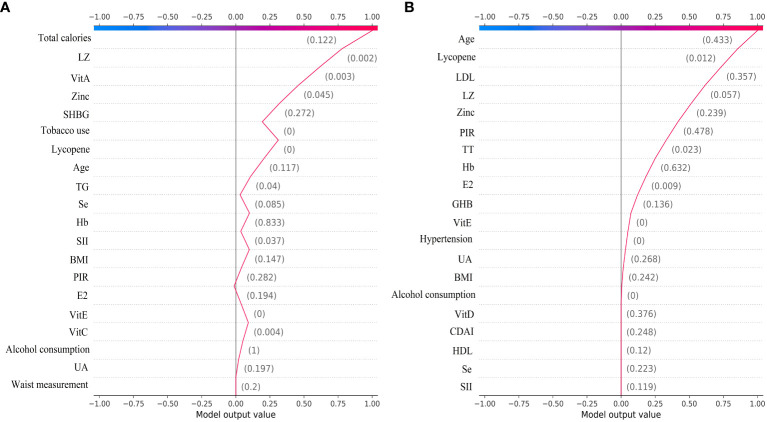
The ML XGBoost model SHAP decision plot. **(A)** Individual decision-making processes in the male group, **(B)** Individual decision-making processes in the female group.

We plotted decision plots revealing the process by which individuals identify gout from a complex XGBoost model. The gray vertical line passing through zero in the decision plot is the baseline of the XGBoost model. The red fold in the decision plot indicates that the individual identification result in the XGBoost model is gout, and the output value for each feature is labeled, along with an indication of whether or not that output value is higher than the average output value. **Figure 8A** illustrates the individual decision-making process of the XGBoost model for the male group. **Figure 8B** shows the individual decision-making process of the XGBoost model for the female group.

**Figure 9:**
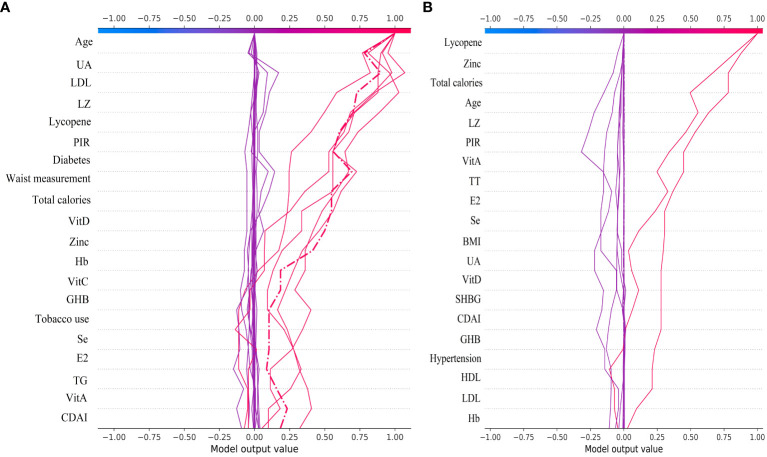
The top 30 individual decision plots for the ML XGBoost model. **(A)** Individual decision-making processes in the male group, **(B)** Individual decision-making processes in the female group.

From the decision plot we can observe that starting from the bottom of the plot, the decision curve shows the final prediction results of the SHAP values in the XGBoost model accumulating from the base value at the bottom to the top, with the final identification boiled down to 0 for no gout prediction and 1 for gout prediction. The decision plot shows the results of the top 30 individual identifications from the XGBoost model, with the red folds being gout predictions and the purple folds being no gout predictions. **Figure 9A** illustrates the individual decision-making process for the top 30 of the XGBoost model for the male group. **Figure 9B** shows the individual decision-making process for the top 30 of the XGBoost model for the female group.

## 4 Discussion

We used an interpretable ML approach to study the relationship between SII, DA, and sex steroid hormones and gout in the NHANES dataset from 2013 to 2016. After comparing the performance, accuracy, and robustness of six ML models, we found that the XGBoost model performed optimally and chose it to identify gout. The use of SHAP summary plots allows for the interpretation of all feature sample predictions and illustrates the relationship between the importance feature of each selected feature in the model, the magnitude of the feature value, and the prediction of gout. Our results suggest that the XGBoost model associated with SII, DA, and sex steroid hormones has superior potential for gout identification. UA was the most important feature in the XGBoost model for both male and female groups. UA levels were negatively correlated with TT, SHBG, CDAI, VitA, and VitC in the male group, and with TT, E2, SHBG, CDAI, VitC, and Zinc in the female group. We also interpreted the individual decision process of the XGBoost model using the SHAP decision plot.

Supervised learning is the most commonly used form of ML in medical research, and this study was based on previous research applying ML algorithms to predict diseases and develop models (27–29). The use of more sophisticated integrated ML algorithms provides higher prediction accuracy and reliability than traditional ML algorithms (30). Therefore we have chosen the traditional learning method SVM, parallel integrated learning method RF, serial integrated learning method GBDT, XGboost, LGBM, and CatBoost respectively to construct the prediction model. Different model discriminant features were also evaluated to select the best performing model for the prediction of gout. The fundamental strategy of clinical research is to identify and address pressing problems in established health areas and to provide preventive solutions for impending diseases. Therefore, it is clinically important for us to use ML algorithms to predict gout based on SII, sex steroid hormones, and DA.

The most important factor in the development of gout is hyperuricemia, and there is a concentration-dependent relationship between the risk of developing gout and serum UA levels (31). Studies have reported that gout increases cardiovascular risk and erectile dysfunction in men (32). Hyperuricemia is associated with erectile dysfunction but not as an independent risk factor for predicting erectile dysfunction (33). A review proposes that the correlation between gout and sexual dysfunction is related to common risk factors, such as joint pain, hypertension, diabetes mellitus, metabolic syndrome, and vascular disease, low-grade chronic inflammation impairs testosterone synthesis and penile vascular endothelial function (34). Our study found a negative correlation between UA level and TT, SHBG, CDAI, VitA, and VitC in males, and the SHAP summary plot suggests that elevated SII and decreased DA intake also increase the risk of gout. Therefore, we hypothesized that decreased TT in males may be associated with gout and that increased DA intake could help prevent gout. In females, we found that UA level was negatively associated with TT, E2, SHBG, CDAI, VitC, and Zinc, while the SHAP summary plot showed that elevated SII increased the risk of gout. It is suggested that decreased E2 and increased inflammatory factors in females may be associated with gout and that increased DA intake may help reduce the risk of gout.

A review suggests that physiological doses of UA are an antioxidant that removes monolinear oxygen and free radicals and reduces protein damage by peroxynitrite (35). In contrast, high concentrations of UA induce oxidative stress and affect lipid synthesis (36). This indicates that oxidative stress mediates the relationship between hyperuricemia and SII. Studies have shown that tumor necrosis factor-α inhibits hepatic production of SHBG by suppressing HNF-4α expression, whereas lipocalin increases HNF-4α expression, and together, they act as homeostatic regulators of SHBG (37). This also fully explains the negative correlation between obesity, gout, and hyperuricemia accompanied by low-grade chronic inflammation and SHBG levels. A review noted that in a prospective study of healthy transgender, serum UA levels were significantly elevated during the female-to-male transition and reduced considerably after the male-to-female change (38). Data from our research express similar results, with serum UA levels varying wildly by gender in the adult U.S. population. SHBG is a glycoprotein produced by the liver with a high affinity for testosterone and a lower relationship for estradiol, and 65% of circulating testosterone binds to SHBG, making SHBG more relevant to men (39). An animal study showed that pioglitazone significantly elevated serum and hepatic SHBG levels, considerably improving triglyceride and total cholesterol levels, insulin resistance, and hyperandrogenemia in rats with polycystic ovary syndrome (40). The causal negative correlation between serum SHBG and metabolism-related diseases provides a theoretical basis for research and development of clinical therapies to increase serum SHBG levels. Still, benefits and risks coexist (41).

In practical medical applications, the advantages of using ML XGBoost models to predict gout far outweigh the challenges. First, the gout prediction model can analyze a large amount of clinical data and complex patterns to provide more accurate gout prediction results, offering the potential for early intervention and reduction of gout prevalence. Second, the XGBoost model can learn and iterate quickly, allowing for continuous model updates and improvements based on newly collected participant data. Third, the ML XGBoost model can automate the processing and analysis of participants’ clinical data, which saves a lot of time cost, and labor. Fourth, despite the powerful automation and intelligence of the ML predictive model, it still faces many challenges when used in practice. The training results of the gout model depend on the quality and reliability of the participant data used, and require significant data computing resources and storage space, especially when computing large-scale participant data.

Our study has some strengths and limitations. First, using the NHANES database ensured a sufficiently large population sample size required for observational studies to make the statistical results more representative. Second, we used the ML method for the prediction of gout with better accuracy and reliability compared to traditional logistic regression methods. Third, We use SHAP to visualize the ML model outputs so that the predictive models have better interpretability. There are also many limitations to our study. Our selection of NHANES data for the diagnosis of gout was based on a questionnaire self-report format, which may have potential diagnostic bias. Because NHANES testing for sex steroid hormones only has data from 2013-2016, data from more cycles could not be selected.

## 5 Conclusion

The interpretable XGBoost ML model demonstrated the best accuracy, efficiency, and robustness in predicting gout based on SII, sex steroid hormones, and DA. Decreased TT in males and decreased E2 in females may be associated with gout, and increased DA intake and decreased SII may reduce the potential risk of gout. We will further conduct continuous tracking analysis and interpretation of the selected features to validate and apply the predictive model for gout identification by extending and updating the database and improving the interpretability of ML.

## Data availability statement

The original contributions presented in the study are included in the article/**Supplementary Material**. Further inquiries can be directed to the corresponding author.

## Ethics statement

The studies involving humans were approved by National Center for Health Statistics (NCHS) Ethics Review Board, Approval No. #2011-17. The studies were conducted in accordance with the local legislation and institutional requirements. The participants provided their written informed consent to participate in this study.

## Author contributions

SC: Data curation, Formal analysis, Writing – review & editing, Conceptualization, Project administration, Supervision, Validation, Visualization. YH: Conceptualization, Funding acquisition, Investigation, Methodology, Resources, Software, Writing – original draft, Writing – review & editing.
