# Supplementary material for: Creating machine learning models that interpretably link systemic inflammatory index, sex steroid hormones, and dietary antioxidants to identify gout using the SHAP (SHapley Additive exPlanations) method
Source: Front Immunol. 2024 May 1;15:1367340. doi: 10.3389/fimmu.2024.1367340 (PMC11094226; doi:10.3389/fimmu.2024.1367340)
Supplement: Supplementary file 1 [file DataSheet_1.pdf]

MEDICAL CONDITIONS – MCQ
Target Group: SPs 1+

MCQ.010 The following questions are about different medical conditions.

Has a doctor or other health professional **ever** told {you/SP} that {you have/s/he/SP has} asthma (**az**-ma)?

CAPI INSTRUCTION:
IF SP AGE >= 16, DISPLAY "YOU" AND "YOU HAVE".
IF SP AGE = 12-15, DISPLAY "SP" AND "S/HE HAS".
IF SP AGE < 12, DISPLAY "YOU" AND "SP HAS".

INTERVIEWER: DO NOT ACCEPT SELF-DIAGNOSED OR DIAGNOSED BY A PERSON WHO IS NOT A DOCTOR OR OTHER HEALTH PROFESSIONAL.

YES 1
NO 2 (MCQ.053)
REFUSED 7 (MCQ.053)
DON'T KNOW 9 (MCQ.053)

HELP SCREEN:
Asthma: Is a disease of the airways that carry air in and out of your lungs. It causes wheezing or whistling sounds when you breathe and can make you short of breath.

MCQ.025 How old {were you/was SP} when {you were/s/he was} **first** told {you/he/she} had asthma (**az**-ma)?

IF LESS THAN 1 YEAR, ENTER 1

CAPI INSTRUCTION:
IF SP AGE >= 16, DISPLAY "WERE YOU" AND "YOU WERE".
IF SP AGE = 12-15, DISPLAY "WAS {SP}" AND "S/HE WAS".
IF SP AGE < 12, DISPLAY "WAS {SP}" AND "YOU WERE".

|___|___|___|
ENTER AGE IN YEARS

CAPI INSTRUCTION:
HARD EDIT: 1-120

REFUSED 77777
DON'T KNOW 99999

MCQ.035 {Do you/Does SP} still have asthma (**az**-ma)?

YES 1
NO 2 (MCQ.053)
REFUSED 7 (MCQ.053)
DON'T KNOW 9 (MCQ.053)

MCQ.040 During the **past 12 months**, {have you/has SP} had an episode of asthma (**az**-ma) or an asthma attack?

YES	1
NO	2
REFUSED	7
DON'T KNOW	9

HELP SCREEN:
Episode/attack: When your asthma symptoms become worse than usual it is called an asthma episode or attack.

MCQ.050 [During the **past 12 months**], {have you/has SP} had to visit an emergency room or urgent care center because of asthma (**az**-ma)?

YES	1
NO	2
REFUSED	7
DON'T KNOW	9

AGQ.030 **During the past 12 months**, {have you/has SP} had an episode of hay fever?

YES	1
NO	2
REFUSED	7
DON'T KNOW	9

HELP SCREEN:
Hay Fever: Hay fever is a collection of symptoms in the nose and eyes, caused by particles of plant pollen in the air. This happens in people who are allergic to these substances. The pollens that cause hay fever vary from person to person and from region to region. Hay fever typically occurs in the Spring, Summer, or Fall when plant pollen is in the air. Examples of plants commonly responsible for hay fever include Trees, Grasses, Flowers, and Ragweed.

MCQ.053 During the **past 3 months**, {have you/has SP} been on treatment for anemia (a-**nee**-me-a), sometimes called "tired blood" or "low blood"? [Include diet, iron pills, iron shots, transfusions as treatment.]

YES	1
NO	2
REFUSED	7
DON'T KNOW	9

HELP SCREEN:
Anemia: Anemia (uh-NEE-me-eh) is a condition in which a person’s blood has a lower than normal number of red blood cells (RBCs).

BOX 2

CHECK ITEM MCQ.055:
IF SP AGE < 6, GO TO END OF SECTION.
IF SP AGE 6-15, GO TO MCQ.082.
IF SP AGE 16+, CONTINUE.

MCQ.070 {Have you/Has SP} ever been told by a doctor or other health care professional that {you/s/he} had psoriasis (sore-eye-asis)?

YES	1	
NO	2	(MCQ 080)
REFUSED	7	(MCQ 080)
DON'T KNOW	9	(MCQ 080)

HELP SCREEN:
Psoriasis: Psoriasis is an itchy red skin rash. It has very sore patches of itchy, thickened red skin with white or silvery scales. It is usually on the elbows, knees, scalp, trunk, hands or feet, but it can be anywhere. It sometimes runs in families.

MCQ.075 {Do you/Does SP} currently have . . .

HAND CARD MCQ1

little or no psoriasis,	1
only a few patches (that could be covered by one or two palms of {your/his/her} hand),	2
scattered patches (that could be covered between three and ten palms of {your/ his/her} hand), or	3
extensive psoriasis (covering large areas of the body, that would be more than ten palms of {your/his/her} hand)?	4
REFUSED	7
DON'T KNOW	9

MCQ.080 Has a doctor or other health professional **ever** told {you/SP} that {you were/s/he/SP was} overweight?

YES	1
NO	2
REFUSED	7
DON'T KNOW	9

BOX 2AA

CHECK ITEM MCQ.079:
IF SP AGE 16-59, GO TO MCQ.082
IF SP AGE 60+, CONTINUE.

MCQ.084 The next question asks about difficulties in thinking or remembering that can make a big difference in everyday activities. This does not refer to occasionally forgetting your keys or the name of someone you recently met. This refers to things like confusion or memory loss that are happening more often or getting worse. We want to know how these difficulties impact {you/SP}. During the past 12 months, {have you/has she/has he} experienced confusion or memory loss that is happening more often or is getting worse?

YES 1
NO 2
REFUSED 7
DON'T KNOW 9

BOX 2A

OMITTED

BOX 3

OMITTED

MCQ.082 Has a doctor or other health professional ever told {you/SP} that {you have/s/he/SP has} celiac (sele-ak) disease, also called sprue (sproo)?

CAPI INSTRUCTION:
IF SP AGE >= 16, DISPLAY "YOU" AND "YOU HAVE".
IF SP AGE = 12-15, DISPLAY "SP" AND "S/HE HAS".
IF SP AGE < 12, DISPLAY "YOU" AND "SP HAS".

YES 1
NO 2
REFUSED 7
DON'T KNOW 9

HELP SCREEN:
Celiac Disease: Is a disease where your bowels and stomach can’t tolerate Gluten. Gluten is a protein found in wheat, rye, and barley flour. When people with this disease eat bread products, it makes them sick.

MCQ.086 {Are you/Is SP} on a gluten-free diet?

YES 1
NO 2
REFUSED 7
DON'T KNOW 9

HELP SCREEN:
A gluten-free diet means not eating foods that contain wheat, rye, and barley.

MCQ.092 {Have you/Has SP} **ever** received a blood transfusion?

YES 1
NO .... 2 (BOX 7)
REFUSED .... 7 (BOX 7)
DON'T KNOW .... 9 (BOX 7)

MCQ.093 In what year did {you/SP} receive {your/his/her} **first** transfusion?

|___|___|___|___|
ENTER 4-DIGIT YEAR

CAPI INSTRUCTION:
HARD EDIT: >= birth year and <= current year

REFUSED 7777
DON’T KNOW 9999

BOX 4

OMITTED

BOX 6

OMITTED

BOX 7

CHECK ITEM MCQ.145:
IF SP'S AGE >= 20, GO TO MCQ.160.
OTHERWISE, CONTINUE.

BOX 7A

CHECK ITEM MCQ.146:
IF SP AGE 8-11 AND SP IS FEMALE, CONTINUE.
OTHERWISE, GO TO MCQ.203.

MCQ.149 Have {SP's} periods or menstrual (**men**-stral) cycles started yet?

YES	1	
NO	2	(MCQ.203)
REFUSED	7	(MCQ.203)
DON'T KNOW	9	(MCQ.203)

HELP TEXT: When a girl starts having periods or menstrual cycles is a very important milestone in growth and development. Growth and development is very related to physical activity and body weight.

MCQ.151 How old was {SP} when she had {her} first menstrual period?

|___|___| YEARS (MCQ.203)

REFUSED	77	(MCQ.203)
DON'T KNOW	99	(MCQ.203)

HARD EDITS: MAXIMUM OF 11 AND AGE OF ONSET MUST BE LESS THAN OR EQUAL TO CURRENT AGE.
SOFT EDIT: IF AGE LESS THAN 7.

HELP TEXT: When a girl starts having periods or menstrual cycles is a very important milestone in growth and development. Growth and development is very related to physical activity and body weight.

BOX 8

OMITTED

BOX 8A

OMITTED

MCQ.160<br>Has a doctor or other health professional **ever** told {you/SP} that {you/s/he} . . .<br><br>CAPI INSTRUCTION:<br>TEXT OF QUESTION SHOULD BE OPTIONAL AFTER FIRST ITEM IS READ.	MCQ.170<br>{Do you/Does SP} still . . . ?	MCQ.180<br>How old {were you/was SP} when {you were/s/he was} **first** told {you/s/he} . . .	MCQ.195<br>Which type of arthritis was it?
a. **had arthritis (**ar-**thry**-tis**)**?<br>YES .... 1 →<br>NO .... 2 (n)<br>REFUSED .... 7 (n)<br>DON'T KNOW..... 9 (n)		had arthritis?<br>|___|___|___| ENTER AGE IN YEARS<br>REFUSED .... 777<br>DON'T KNOW... 999	Osteoarthritis or degenerative arthritis.......... 1<br>Rheumatoid arthritis.................................... 2<br>Psoriatic arthritis .... 3<br>Other.. 4<br>REFUSED... 7<br>DON'T KNOW... 9
n. **had gout**?<br>YES .... 1 →<br>NO .... 2 (b)<br>REFUSED .... 7 (b)<br>DON'T KNOW..... 9 (b)		had gout?<br>|___|___|___| ENTER AGE IN YEARS<br>REFUSED .... 777<br>DON'T KNOW... 999	
b. **had congestive heart failure**?<br>YES .... 1 →<br>NO .... 2 (c)<br>REFUSED .... 7 (c)<br>DON'T KNOW..... 9 (c)		had congestive heart failure?<br>|___|___|___| ENTER AGE IN YEARS<br>REFUSED .... 777<br>DON'T KNOW... 999	
c. **had coronary (kor**-o-nare-ee**) heart disease**?<br>YES .... 1 →<br>NO .... 2 (d)<br>REFUSED .... 7 (d)<br>DON'T KNOW..... 9 (d)		had coronary heart disease?<br>|___|___|___| ENTER AGE IN YEARS<br>REFUSED .... 777<br>DON'T KNOW... 999	
d. **had angina (**an-**gī**-na**), also called angina pectoris**?<br>YES .... 1 →<br>NO .... 2 (e)<br>REFUSED .... 7 (e)<br>DON'T KNOW..... 9 (e)		had angina, also called angina pectoris?<br>|___|___|___| ENTER AGE IN YEARS<br>REFUSED .... 777<br>DON'T KNOW... 999	

e. **had a heart attack (also called myocardial infarction (**my-O-**car**-dee-al in-**fark**-shun**))**?<br><br>YES .... 1 →<br>NO...................... 2 (f)<br>REFUSED .... 7 (f)<br>DON'T KNOW..... 9 (f)		had a heart attack (also called myocardial infarction)?<br><br>|___|___|___| ENTER AGE IN YEARS<br><br>REFUSED .... 777<br>DON'T KNOW.. 999	
f. **had a stroke**?<br><br>YES .... 1 →<br>NO...................... 2 (g)<br>REFUSED .... 7 (g)<br>DON'T KNOW..... 9 (g)		had a stroke?<br><br>|___|___|___| ENTER AGE IN YEARS<br><br>REFUSED .... 777<br>DON'T KNOW.. 999	
g. **had emphysema (**emph-phi-**see**-ma**)**?<br><br>YES .... 1 →<br>NO...................... 2 (m)<br>REFUSED .... 7 (m)<br>DON'T KNOW..... 9 (m)		had emphysema?<br><br>|___|___|___| ENTER AGE IN YEARS<br><br>REFUSED .... 777<br>DON'T KNOW.. 999	
m. had a thyroid (**thigh**-roid) problem?<br><br>YES .... 1 →<br>NO...................... 2 (k)<br>REFUSED .... 7 (k)<br>DON'T KNOW..... 9 (k)	have a thyroid problem?<br><br>YES..................... 1<br>NO....................... 2<br>REFUSED .... 7<br>DON'T KNOW 9	had a thyroid problem?<br><br>|___|___|___| ENTER AGE IN YEARS<br><br>REFUSED .... 777<br>DON'T KNOW.. 999	
k. **had chronic bronchitis**?<br><br>YES .... 1 →<br>NO...................... 2 (l)<br>REFUSED .... 7 (l)<br>DON'T KNOW..... 9 (l)	have chronic bronchitis?<br><br>YES..................... 1<br>NO....................... 2<br>REFUSED .... 7<br>DON'T KNOW 9	had chronic bronchitis?<br><br>|___|___|___| ENTER AGE IN YEARS<br><br>REFUSED .... 777<br>DON'T KNOW.. 999	
l. **had any kind of liver condition**?<br><br>YES ....1 →<br>NO..................2 (MCQ.160o)<br>REFUSED ......7 (MCQ.160o)<br>DON'T KNOW .9 (MCQ.160o)	have this liver condition?<br><br>YES..................... 1<br>NO....................... 2<br>REFUSED .... 7<br>DON'T KNOW 9	had this liver condition?<br><br>|___|___|___| ENTER AGE IN YEARS<br><br>REFUSED .... 777<br>DON'T KNOW.. 999	

MCQ.160o. had COPD?<br><br>YES .... 1 →<br>NO .... 2<br>REFUSED...... 7<br>DON'T KNOW 9			

HELP SCREENS FOR MCQ.160

MCQ160a

Arthritis: Is a disease that causes pain, swelling or stiffness in joints, for example the hand, the knee, or neck. Common kinds of arthritis are osteoarthritis and rheumatoid arthritis.

MCQ.195

Osteoarthritis: Is the most common kind of arthritis older persons. It is also called degenerative joint disease. Most often, it affects the knees, the hips, the hands, the feet, and the spine. There is usually bony joint enlargement. There can be joint deformity or pain.

Rheumatoid Arthritis: Causes inflammation, redness and swelling of both hands and knees, but it can affect joints anywhere in the body. You may feel sick and tired, and sometimes there are fevers.

Psoriatic Arthritis: Is arthritis caused by the skin rash Psoriasis. Most often it causes redness and swelling of joints such as the spine, knees, hips and hands.

Arthritis: Is a disease that causes pain, swelling or stiffness in joints, for example the hand, the knee, or neck. Common kinds of arthritis are osteoarthritis and rheumatoid arthritis.

MCQ160n

Gout: Gout attacks are the sudden onset of pain, redness and swelling in a joint. The big toe is the most common joint attacked, but knee and wrist attacks are also common. Gout is caused by uric acid crystal build up in the body.

MCQ160b

Congestive Heart Failure: Is when the heart can't pump enough blood to the body. Blood and fluid "back up" into the lungs, which makes you short of breath. Heart failure causes fluid buildup in and swelling of the feet, legs and ankles.

INTERVIEWER: DO NOT COUNT HEART MURMURS, IRREGULAR HEART BEATS, CHEST PAIN OR HEART ATTACKS.

MCQ160c

Coronary Heart Disease: Is when the blood vessels that bring blood to the heart muscle become narrow and hardened due to plaque (plak). Plaque buildup is called atherosclerosis (ATH-er-o-skler-O-sis). Blocked blood vessels to the heart can cause chest pain or a heart attack.

INTERVIEWER: IF THE RESPONDENT REPORTS CHEST PAIN, PROBE IF A DOCTOR TOLD THEM THAT THEY HAD BLOCKED BLOOD VESSELS OR CORONARY HEART DISEASE.

MCQ160d

Angina (Angina Pectoris): (AN-ji-na or an-JI-na). Angina is chest pain or discomfort that occurs when the heart does not get enough blood.

INTERVIEWER: IF THE RESPONDENT REPORTS CHEST PAIN, PROBE IF A DOCTOR TOLD THEM THAT THEY HAD BLOCKED BLOOD VESSELS OR ANGINA.

MCQ160e

Heart Attack (Myocardial Infarction): A heart attack happens when there is narrowing of a blood vessel that supplies the heart. A blood clot can form and suddenly cut off the blood supply to the heart muscle. This damage

causes crushing chest pain that may also be felt in the arms or neck. There can also be nausea, sweating, or shortness of breath.

MCQ160f

Stroke: Is when the blood supply to a part of the brain is suddenly cut off by a blood clot or a burst blood vessel in the brain. The part of the brain affected can no longer do its job. There can be numbness or weakness on one side of the body; trouble speaking or understanding speech; loss of eyesight; trouble with walking, dizziness, loss of balance or coordination; or severe headache.

MCQ160g

Emphysema: Is disease where the tiny air sacs in the lungs become damaged so less air goes in and out. As a result, the body does not get the oxygen it needs. Emphysema makes it hard to catch your breath. It is often due to smoking.

.

MCQ160m

Thyroid Problem: The thyroid is a gland in the neck that makes thyroid hormone. The thyroid sets your body's energy level: the temperature and heart rate. Thyroid problems include thyroid levels that are too high or too low, an inflamed or enlarged gland, and thyroid lumps or cancer.

INTERVIEWER: INCLUDE HYPERTHYROID (OVERACTIVE THYROID); HYPOTHYROID (UNDERACTIVE THYROID); GRAVES DISEASE (HYPERTHYROID AND/OR THYROID EYE DISEASE); HASHIMOTO'S THYRODITIS (INFLAMED THYROID); POSTPARTUM THYROIDITIS (INFLAMED THYROID THAT HAPPENS AFTER DELIVERY OF A BABY); GOITER (ENLARGED THYROID); THYROID NODULE (LUMP IN THYROID-NOT CANCER); AND THYROID CANCER.

MCQ160k

Chronic Bronchitis: Is a long lasting breathing problem where you constantly cough up phlegm. Often there is a daily cough with phlegm for several months at a time for two or more years and you are short of breath. It is often due to smoking.

MCQ.160o

COPD: stands for “Chronic Obstructive Pulmonary Disease.” It includes both Emphysema and Chronic Bronchitis. It is lung problem where you have trouble getting air in and out of your lungs. You may also have constant cough and phlegm.

MCQ.203 Has **anyone ever** told {you/SP} that {you/she/he/SP} had yellow skin, yellow eyes or jaundice? Please do not include infant jaundice, which is common during the first weeks after birth.

CAPI INSTRUCTION:
IF SP AGE >= 16, DISPLAY “YOU” AND “YOU”.
IF SP AGE = 12-15, DISPLAY "SP" AND “S/HE”.
IF SP AGE = 6-11, DISPLAY “YOU” AND “SP”.

INTERVIEWER: <u>DO</u> ACCEPT SELF-DIAGNOSED OR DIAGNOSED BY A PERSON WHO IS NOT A DOCTOR OR OTHER HEALTH PROFESSIONAL.

YES 1
NO .... 2 (BOX 8B)
REFUSED .... 7 (BOX 8B)
DON'T KNOW .... 9 (BOX 8B)

HELP SCREEN:
Infant jaundice is a yellow discoloration in a newborn baby’s skin and eyes.

MCQ.206 How old {were you/was SP} when {you were/s/he was} **first** told {you/s/he} had yellow skin, yellow eyes or jaundice?

INTERVIEWER: IF LESS THAN 1 YEAR, ENTER 0

CAPI INSTRUCTION:
IF SP AGE >= 16, DISPLAY "WERE YOU" AND "YOU WERE" AND “YOU”.
IF SP AGE = 12-15, DISPLAY "WAS {SP}" AND "S/HE WAS" AND “S/HE”.
IF SP AGE = 6-11, DISPLAY "WAS {SP}" AND "YOU WERE" AND “S/HE”.

HARD EDIT: MCQ.206 > SP AGE

|___|___|___|
ENTER AGE IN YEARS

CAPI INSTRUCTION:
HARD EDIT: 0-120

REFUSED 777
DON'T KNOW 999

HELP SCREEN:
Infant jaundice is a yellow discoloration in a newborn baby’s skin and eyes.

BOX 8AA

CHECK ITEM MCQ.209:
IF SP MCQ.206 = ZERO, CONTINUE.
ELSE, GO TO BOX 8B.

MCQ.207 Please remember not to include infant jaundice, which is common during the first weeks after birth. {Have you/Has SP} been told that {you/he/she} had yellow skin, yellow eyes or jaundice other than during the first weeks after birth?

YES	1	(BOX 8B)
NO	2	
REFUSED	7	(BOX 8B)
DON'T KNOW	9	(BOX 8B)

CAPI INSTRUCTION:
IF MCQ.207 = NO, THEN CHANGE MCQ.203 = NO AND MCQ.206 = EMPTY AND CONTINUE TO BOX 8B.

BOX 8B

CHECK ITEM MCQ.208:
IF SP AGE 6-19, GO TO MCQ300b
IF SP AGE ≥ 20, CONTINUE.

MCQ.220 {Have you/Has SP} **ever** been told by a doctor or other health professional that {you/s/he} had cancer or a malignancy (ma-**lig**-nan-see) of any kind?

YES	1	
NO	2	(MCQ.300a)
REFUSED	7	(MCQ.300a)
DON'T KNOW	9	(MCQ.300a)

HELP SCREEN:
Cancer: Is an abnormal growth that can spread to other parts of the body. This causes damage and even death. Most cancers are named for where they start: for example lung cancer or breast cancer. A cancer is also called a "malignancy" or a "malignant tumor".

Malignancy: A tumor or growth that is a cancer. (see Cancer)

MCQ.230 What kind of cancer was it?

ENTER UP TO 3 KINDS. IF RESPONDENT OFFERS MORE THAN 3, ENTER 66 AS THE 4TH RESPONSE.

CAPI INSTRUCTIONS:
ALLOW UP TO 3 ENTRIES.
ALLOW 'MORE THAN 3 KINDS (CODE 66) ONLY AS 4TH ENTRY.

( ) ( ) ( ) ( )

BLADDER	10	LEUKEMIA	21	SKIN (NON-MELANOMA)	32
BLOOD	11	LIVER	22	SKIN (DON'T KNOW WHAT KIND)	33
BONE	12	LUNG	23	SOFT TISSUE (MUSCLE OR FAT)	34
BRAIN	13	LYMPHOMA/HODGKINS' DISEASE	24	STOMACH	35
BREAST	14	MELANOMA	25	TESTIS (TESTICULAR)	36
CERVIX (CERVICAL)	15	MOUTH/TONGUE/LIP	26	THYROID	37
COLON	16	NERVOUS SYSTEM	27	UTERUS (UTERINE)	38
ESOPHAGUS (ESOPHAGEAL)	17	OVARY (OVARIAN)	28	OTHER	39
GALLBLADDER	18	PANCREAS (PANCREATIC)	29	MORE THAN 3 KINDS	66

KIDNEY .... 19<br>LARYNX/WINDPIPE 20	PROSTATE .... 30<br>RECTUM (RECTAL) 31	REFUSED .... 77<br>DON'T KNOW 99

BOX 9

LOOP 1:
ASK MCQ.240 FOR EACH TYPE OF CANCER (CODES 10-39 AND CODE 99) ENTERED IN MCQ.230.

MCQ.240 How old {were you/was SP} when {TYPE OF CANCER/cancer} was **first** diagnosed?

CAPI INSTRUCTIONS:
DISPLAY TYPE OF CANCER (CODE 10-39) ENTERED IN MCQ.230.
DISPLAY "CANCER " IF DON'T KNOW ENTERED IN MCQ.230.

|___|___|___|
ENTER AGE IN YEARS

REFUSED 777
DON'T KNOW 999

BOX 9A

END LOOP 1:
ASK MCQ.240 FOR NEXT TYPE OF CANCER (CODES 10-39 AND CODE 99) ENTERED IN MCQ.230.
IF NO NEXT TYPE, CONTINUE WITH MCQ.300a.

BOX 10

OMITTED

BOX 10A

OMITTED

MCQ.300 a/b/c Including living and deceased, **were any of {SP's/your} close biological** that is, blood **relatives** including father, mother, sisters or brothers, **ever told by a health professional that they had** . . .

CAPI INSTRUCTION:
TEXT OF QUESTION SHOULD BE OPTIONAL, "[ ]'S, AFTER FIRST TIME.

a. **a heart attack or angina (**an-**gī**-na**) before the age of 50?**

YES	1
NO	2
REFUSED	7
DON'T KNOW	9

b. **asthma (az**-ma**)?**

CAPI INSTRUCTION:
IF SP AGE 6-19, DISPLAY: Including living and deceased, were any of {SP's/your} close biological that is, blood relatives including father, mother, sisters or brothers, ever told by a health professional that they had . . .

YES	1
NO	2
REFUSED	7
DON'T KNOW	9

BOX 10c

OMITTED

BOX 10D

CHECK ITEM MCQ.360:
IF SP AGE 6-15, GO TO END OF SECTION.
IF SP AGE16-19, GO TO MCQ.365.
OTHERWISE, CONTINUE.

c. **diabetes?**

YES	1
NO	2
REFUSED	7
DON'T KNOW	9

BOX 11

OMITTED

MCQ.365 a/b/c/d

To lower {your/SP's} risk for certain diseases, during the past 12 months {have you/has s/he} ever been told by a doctor or health professional to:

RESPONSES: YES = 1, NO = 2, REFUSED = 7, DON'T KNOW = 9

a. control {your/his/her} weight or lose weight? ____

b. increase {your/his/her} physical activity or exercise? ____

c. reduce the amount of sodium or salt in {your/his/her} diet? ____

d. reduce the amount of fat or calories in {your/his/her} diet? ____

HELP SCREEN: Controlling your weight might be recommended to help prevent high blood pressure, diabetes, high cholesterol and other conditions.

MCQ.370 a/b/c/d

To lower {your/his/her} risk for certain diseases, {are you/is s/he} now doing any of the following:

RESPONSES: YES = 1, NO = 2, REFUSED = 7, DON'T KNOW = 9

a. controlling {your/his/her} weight or losing weight? ____

b. increasing {your/his/her} physical activity or exercise? ____

c. reducing the amount of sodium or salt in {your/his/her} diet? ____

d. reducing the amount of fat or calories in {your/his/her} diet? ____

HELP SCREEN: Controlling your weight might be recommended to help prevent high blood pressure, diabetes, high cholesterol and other conditions.

BOX 12

CHECK ITEM MCQ.375:
IF SP AGE < 60, GO TO END OF SECTION.
OTHERWISE, CONTINUE.

MCQ.380

During the **past 7 days**, how often {have you/has SP} had trouble remembering where {you/he/she} put things, like {your/his/her} keys or {your/his/her} wallet? Would you say....

Never..	0
About once	1
Two or three times.......................................	2
Nearly every day..	3
Several times a day......................................	4
REFUSED	7
DON'T KNOW	9
